# Impact of the COVID-19 Pandemic on Radiotherapy Supply

**DOI:** 10.1155/2021/5550536

**Published:** 2021-05-05

**Authors:** Francesco Tramacere, Artor Niccoli Asabella, Maurizio Portaluri, Corinna Altini, Cristina Ferrari, Lilia Bardoscia, Angela Sardaro

**Affiliations:** ^1^Radiation Oncology, Department of Radiotherapy, O. Policlinic “A. Perrino”, Strada Statale 7 per Mesagne, Brindisi 72100, Italy; ^2^Nuclear Medicine Unit, A. O. Policlinic “A. Perrino”, Strada Statale 7 per Mesagne, Brindisi 72100, Italy; ^3^Nuclear Medicine Unit, Interdisciplinary Department of Medicine, University of Bari Aldo Moro, Piazza Giulio Cesare 11, Bari 70124, Italy; ^4^Radiotherapy Unit, Clinical Cancer Centre, AUSL-IRCCS, Reggio Emilia, Italy; ^5^Section of Radiology and Radiation Oncology, Interdisciplinary Department of Medicine, University of Bari Aldo Moro, Piazza Giulio Cesare 11, Bari 70124, Italy

## Abstract

**Background:**

The impetuous entrance of the COVID-19 pandemic in Italy in March 2020, after the onset and diffusion in China, found the health system widely unfit to face the large amount of infected patients. The matter of this investigation was to evaluate how pandemic fear and guidelines for limiting the diffusion of SARS-CoV-2 virus could have impacted the regular supply of radiotherapy (RT) and the outcome of the treatments.

**Materials and Methods:**

From March 9, 2020, to May 29, 2020, a register has been established to record patients that cancelled or postponed the RT appointment. The reasons were as follows: (1) patients whose appointments were postponed by the staff according to national guidelines; (2) patients who asked themselves to postpone the appointment; (3) patients who interrupted the treatment for causes directly or indirectly related to the pandemic; (4) patients who cancelled their care path.

**Results:**

A total number of 277 patients started regular RT, and 384 respected their computed tomography (CT) simulation appointment, but 60 of them had alteration of their therapeutic pathway. Among these, 18 cancelled their appointment. 42 patients asked to postpone their procedure. Twenty-seven out of 42 adduced directly or indirectly SARS-CoV-2 infection-related reasons.

**Conclusions:**

The COVID-19 pandemic affected the regular RT delivery to oncologic patients, owing to the delay or cancellation of procedures with the likely effect to observe worsening of local disease control and reduced survival rates in the future.

## 1. Introduction

At the onset of the COVID-19 pandemic in Italy, i.e., at the beginning of March 2020, radiotherapy (RT) treatments were included in the list of the specialties which had to be guaranteed to all patients with cancer for whom there was indication and they should not have been canceled, contrary to what happened for other medical procedures considered nonurgent [1]. This decision was due to the shortage of resources of personnel which imposed many wards to be converted into coronavirus disease-19 (COVID-19) wards, with the aim to provide beds and adequate healthcare to the extremely increasing number of patients with SARS-CoV-2 symptomatic infection. Nonetheless, recommendations from the national scientific society Italian Association of Radiotherapy and Clinical Oncology (AIRO) suggested to postpone some nonurgent and deferrable RT treatments for patients with better prognosis (e.g., breast and prostate cancer) and evaluate the ratio risk/benefit in each case; to prefer hypofractionation schemes where indicated; and to use pharmacological treatments at home for the relief of symptoms of similar efficacy of radiotherapy. Furthermore, such guidelines suggested not to start treatment in SARS-CoV-2-positive patients and to stop it if the patients became symptomatic or positive during RT delivery. In this last case, the recommendations suggest to evaluate each case in order to complete or not the treatment, and in affirmative decision, it could happen through the permission of Health Authority, a high-level protection of the staff, and careful disinfection of the treatment room [2]. This hypothesis is not supported by any scientific evidence.

During the COVID-19 pandemic, consultations were conducted by means of telematic tools, and follow-up visits were postponed for patients who declared themselves as without evidence of oncologic disease in order to minimize patients' exposure to viral diffusion without compromising oncological outcomes. In any case, the COVID-19 pandemic produced the cancellation of appointments, postponing of procedures, and treatments' interruption. We described this phenomenon in a Radiation Oncology Department in Southern Italy. The matter of the investigation was to evaluate the impact of pandemic fear or guidelines for limiting the diffusion of SARS-CoV-2 infection on the regular supply of radiotherapy and treatment outcomes.

## 2. Materials and Methods

**Figure 1:**
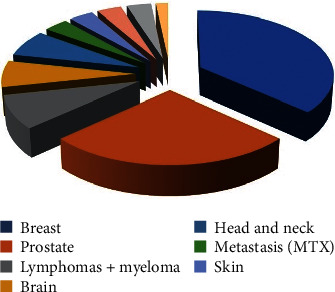
Diagnosis distribution.

From March 9, 2020, to May 29, 2020, a total number of 277 cancer patients started RT as regularly programmed, and 384 patients respected their planning CT scan appointment, but 60 patients changed their therapeutic pathway. The characteristics of the recorded patients are reported in Table 1 and Figure 1.

From the start of COVID-19 rapid spread in Italy, since the enactment of the Italian Government official recommendation statements (reference), and once AIRO tips for the management of oncological patients in the context of the COVID-19 pandemic were available [2], a register has been established to record patients who cancelled or delayed their RT appointment. Anthropometric data (sex and age), information on cancer (time of diagnosis, histology, stage of disease, and therapeutic approach—multimodal or RT alone), intent of RT (radical vs. adjuvant vs. neoadjuvant), association with pharmacological therapy, phase of the RT program involved (first access and/or CT simulation and/or RT start and/or RT delivery), the reasons for postponing or refusing the appointment, and the time of the delay request were collected.

The motivation for postponing/refusing the RT appointment was then gathered in 3 different groups: (1) delay disposed by the radiation oncologist in accordance with national and international principles and recommendations; (2) delay for patient's request; (3) delay or interruption directly or indirectly due to COVID-19 infection.

The RT workflow consisted in treatment decision during the first access of patients at the Radiation Oncology Department, followed by CT simulation, treatment planning, and plan approval with quality assurance, and finally in treatment delivery. The definition of the treatment program and informed consent, CT simulation, and RT delivery required patient presence. We did not postpone the first consultations (treatment decision phase) for patients who needed definitive, immediate treatment for symptomatic, extensive, and/or locally advanced malignancies. We considered the delay for some CT simulations and nonurgent or deferral treatments such as localized, low-risk prostate cancer or patients with intermediate-high-very high risk disease undergoing androgen deprivation therapy, adjuvant RT for early stage, hormone-sensitive breast cancer, and those for adjuvant purposes in general, especially for elderly patients, always trying to respect the timing indicated by international guidelines and recommendations. The appointment scheduling for visits and planning CT scan were scattered across the day to minimize the number of people present in the waiting rooms at the same time [3, 4].

## 3. Results

From March 9, 2020, to May 29, 2020, 721 cancer patients in total were listed in our dedicated register. Among these, 277 patients started RT as regularly programmed, 384 patients respected their planning CT scan appointment, but 60 patients changed their therapeutic pathway. The characteristics of the recorded patients are reported in Table 1.

Eighteen (30%) out of 60 patients cancelled their appointment (Table 2), while 42 (70%) of them postponed the programmed radiation treatment (Table 3).

Among the former group, 6 (10%) patients were recruited for palliative treatment, and the remaining 12 (20%) were candidates for radical (two, 3.3%) and adjuvant (ten, 16.7%) RT, respectively.

Tables 2 and 3 also provide the intent of RT prescription per patient, sex, age, and the reason for the delay. Median age was slightly higher in the subgroup of RT cancellation than in the RT delay group (72 vs. 68 years).

When adjusted for patients' sex, median age was 63 years for women and 68 years for men. Patients candidates to adjuvant RT were the most represented in both the aforementioned subgroups (32 53%). Diagnosis of breast and prostate cancer was the most frequent (38/60, 63%) (Table 4).

Motivation 3 (COVID-19-related delay or interruption) was found to be the most frequent reason for procedure delay or interruption (27 vs. 15) (Table 3). In detail, such motivations consisted in fever at the triage point at the entrance of the hospital, positive or ongoing SARS-CoV-2 test for suspected infections, positive SARS-CoV-2 antibody serologic test, positive tests in relatives, and subsequent quarantine. Moreover, among prostate cancer patients, delay of staging or restaging choline PET/CT performance was a further cause of appointment rescheduling. Stratification of RT procedures for oncological intent showed that median time interval of delay was substantially equivalent among the selected subgroups, with an overall median delay interval of 27.5 days (range: 4–126) and median time to delay of 25 days (range: 4–74) for radical RT and 30 days (range: 5–112 and 131–126, respectively) for adjuvant and palliative RT (Table 5).

In this setting, motivation 3 was associated with a median 24 days (range: 4–126) of delay, compared to 37 days (range: 30–60) for motivation 1 (radiation oncologist decision) and 30 days (range: 10–82) for motivation 2 (patients' request). The postponing request involved mostly the date of CT-simulation procedure (26/42, 60%), with 15 (40.6%) cases in the adjuvant RT subgroup.

## 4. Discussion

The impetuous entrance of the COVID-19 pandemic in Italy in March 2020, after the onset and diffusion in China, found the health system largely unfit to face the large amount of infected patients [4–6]. Although social distancing and the need for staff preservation to avoid severe shortage by SARS-CoV-2 infection and related impairment of the full functioning of radiation therapy facilities were recommended in national guidelines and international praxis adopted in Asiatic countries, at the onset of the pandemic in the radiotherapy department, some authors reminded correctly that “suboptimal delivery of radiotherapy (including delays, interruptions, or omissions) has been demonstrated to compromise both local control and survival.” [7,8] They further recalled that “the findings of a systematic review demonstrate that delaying the initiation of adjuvant radiotherapy >8 weeks after surgery doubles the risk of local recurrence in patients with breast cancer. Furthermore, a meta-analysis conducted by the Early Breast Cancer Trialists' Collaborative Group indicates that radiotherapy reduces the risk of local recurrence, with 3.8% absolute reduction in 15-year risk of breast cancer mortality (from 25.2% to 21.4%; *P*=0.00005).” [9]

It can be expected for sure that the median delay of 30 days we registered in the present observational study, which should be added to the waiting time generally registered in Italy between the first evaluation and the start of RT, will produce, in the medium/long term, adverse effects to the patients in terms of worse local control of the disease and lower survival rates. This effect cannot be measured at the moment, but the large amount of literature reporting the negative impact of longer treatment time and waiting time in radiotherapy is well known. Because the majority of the patients performed an adjuvant treatment for breast tumor (22/60) or a radical one for prostate tumor (16/60), which are cancer diseases with low mortality rate and need long time to develop a local recurrence, it is impossible to evaluate the impact on the outcome of the delay in this small group of patients.

In our series, there was a shorter postponing time for radical patients compared to those presenting for adjuvant or palliative irradiation because of the urgency of the need for RT start. The adjuvant patients were the majority in the postponing group, likely because their care pathway had been ensured in part by the surgical time. This is in line with the American Society for Radiation Oncology (ASTRO) and the European Society for Radiotherapy and Oncology (ESTRO) consensus statements for risk-adapted RT strategies during the COVID-19 pandemic, with the aim to protect patients and healthcare professionals from potential exposure and at the same time ensure high-quality treatments both for saving lives and for palliative purposes [11].

It is noteworthy that the median age of patients who refused or required to postpone RT was higher (72 vs. 68 years) than the overall series. Such findings support that the fear of SARS-CoV-2 infection was widely spread in the elder population.

In the analyzed group, 18/60 (30%) patients refused to start treatment, and to date, we had no further updates concerning their medical choices since they were not traceable either by the authorized contact person. Such findings were cause for concern, especially for the two (10%) patients needing for radical treatment. We supposed the “palliative” group was likely to be managed at home with the palliative medical approach, instead. Among the motivations attributed to the delay or refusal of RT, some of them regarded the waiting for staging or restaging diagnostic procedures, such as choline-F PET/CT for prostate cancer patients, propaedeutic to the start of primary or postoperative RT on the prostate/prostate bed, rather than ablative, stereotactic RT for nodal and/or bone oligometastases. It is well known that, during the lockdown, many medical activities were performed only on an emergency basis although the governmental provisions established that oncologic patients had a priority in the diagnostic and therapeutic pathway, but this could not be observed because the pandemic absorbed a large part of technological and human resources.

## 5. Conclusion

Our findings seemed to confirm that different reasons, directly or indirectly related to the COVID-19 pandemic, impacted on the regular supply of radiotherapy, determining delay or cancellation of procedures with the likely effect to observe, in the future, worse local control of the disease and survival rates in these oncological patients.

## Figures and Tables

**Table 1:** Description of patients' characteristics.

	M	F	M + F
Sex	28	32	60
Median age	69 years (50–87)	64 years (50–87)	68 years (29–87)
Delay	21	21	42
Refuse	7	11	18
Radical	12	4	17
Adjuvant	9	23	32
Palliative	6	5	11

**Table 2:** Description of patients who refused radiotherapy.

	M	F	M + F
Median age	72 years (52–86)	72 years (46–84)	72 years (46–84)
Radical	2	-	2
Adjuvant	2	8	10
Palliative	3	3	6
Motivation 1	3	-	3
Motivation 2	2	5	7
Motivation 3	2	6	8

**Table 3:** Description of patients who delayed radiotherapy.

	M	F	M + F
Median age	68 years (50–87)	60 years (29–86)	63 years (20–87)
Radical	11	4	15
Adjuvant	7	15	22
Palliative	3	2	5
Motivation 1	3	0	3
Motivation 2	5	7	12
Motivation 3	13	14	27

**Table 4:** Oncologic diagnosis of patients.

Breast	22
Prostate	16
Lymphomas + myeloma	5
Brain	4
Head and neck	4
Metastasis (MTX)	2
Skin	2
Bladder	2
Gynecological	2
Lung	1

**Table 5:** Mean time of delay in patients with the postponed procedure according to the intent of treatment and motivation.

	Median (min–max) days of delay
Radical	25 (4–74)
Adjuvant	30 (5–112)
Palliative	30 (13–126)
Motivation 1	37 (30–60)
Motivation 2	30 (10–82)
Motivation 3	24 (4–126)
Overall	27.5 (4–126)

## Data Availability

The data used to support the findings of this study are restricted in order to protect patients' privacy. The data are available from Dr. Maurizio Portaluri (m.portaluri@asl.brindisi.it; Chief of the Radiation Oncology, Department of Radiotherapy, O. Policlinic “A. Perrino,” Brindisi) who meet the criteria for access to confidential data.

## References

[1] Ministero della Salute–Allegato 1-Raccomandazioni per la gestione dei pazienti oncologici e onco-ematologici in corso di emergenza da COVID-19, 2020, http://www.salute.gov.it/imgs/C_17_pagineAree_5373_3_file.pdf

[2] Associazione Italiana di Radioterapia Oncologica. Documento di indirizzo per la valutazione e la gestione del rischio dei pazienti e degli operatori nei reparti di radioterapia oncologica in corso di diffusione del COVID-19, 2020, https://www.radioterapiaitalia.it/wp-content/uploads/2020/03/v-2-Documento-AIRO-COVID-19-24-03-2020.pdf

[3] Krengli M., Ferrara E., Mastroleo F., Brambilla M., Ricardi U. (2020). Running a radiation Oncology department at the time of coronavirus: an Italian experience. *Advances in Radiation Oncology*.

[4] Portaluri M., Tramacere F., Portaluri T., Gianicolo E. A. L. (2020). Southern Italy: how the availability of radiation therapy, patient outcomes, and risk to health care providers have changed during the coronavirus disease 2019 pandemic. *Advances in Radiation Oncology*.

[5] Filippi A. R., Russi E., Magrini S. M., Corvò R. (2020). Letter from Italy: first practical indications for radiation therapy departments during COVID-19 outbreak. *International Journal of Radiation Oncology ∗ Biology ∗ Physics*.

[6] Portaluri M., Bambace S., Tramacere F., Errico A., Carbone S., Portaluri T. (2020). Staff and patient protection in radiation Oncology departments during coronavirus disease 2019 (COVID-19) pandemic. *Advances in Radiation Oncology*.

[7] Wu S., Zheng D., Liu Y., Hu D., Wei W., Han G. (2020). Radiation therapy care during a major outbreak of COVID-19 in Wuhan. *Advances in Radiation Oncology*.

[8] Nagar H., Formenti S. C. (2020). Cancer and COVID-19-potentially deleterious effects of delaying radiotherapy. *Nature Reviews Clinical Oncology*.

[9] Huang J., Barbera L., Brouwers M., Browman G., Mackillop W. J. (2003). Does delay in starting treatment affect the outcomes of radiotherapy? A systematic review. *Journal of Clinical Oncology*.

[10] Darby S., Darby S., McGale P (2011). Effect of radiotherapy after breast-conserving surgery on 10-year recurrence and 15-year breast cancer death: meta-analysis of individual patient data for 1001 women in 17 randomised trials. *Lancet*.

[11] Thomson D. J., Palma D., Guckenberger M. (2020). Practice recommendations for risk-adapted head and neck cancer radiotherapy during the COVID-19 pandemic: an ASTRO-ESTRO consensus statement. *Radiotherapy and Oncology*.

